# Fabrication of Mid-Infrared Porous Anodic Alumina Optical Microcavities via Aluminum Anodization

**DOI:** 10.3390/ma17225620

**Published:** 2024-11-18

**Authors:** Ewelina Białek, Weronika Gruszczyńska, Maksymilian Włodarski, Malwina Liszewska, Małgorzata Norek

**Affiliations:** 1 Institute of Materials Science and Engineering, Faculty of Advanced Technologies and Chemistry, Military University of Technology, Str. Gen. Sylwestra Kaliskiego 2, 00-908 Warsaw, Poland; ewelina.bialek@wat.edu.pl (E.B.);; 2 Institute of Optoelectronics, Military University of Technology, Str. Gen. Sylwestra Kaliskiego 2, 00-908 Warsaw, Poland; maksymilian.wlodarski@wat.edu.pl (M.W.); malwina.liszewska@wat.edu.pl (M.L.)

**Keywords:** anodization, porous anodic alumina (PAA), photonic crystals, optical microcavity, mid-infrared (MIR)

## Abstract

This study reports the production of mid-infrared (MIR) porous anodic alumina (PAA)-based microcavities with tunable optical quality. The spectral position of the cavity resonance peak (λ_C_), along with its intensity (I_R_) and Q-factor, varies depending on the geometric positioning of the cavity layer within the multilayer stack of alternating low- and high-porosity layers, as well as the type of cavity produced—either by high voltage (Cv_H_-type) or low voltage (Cv_L_-type) pulses. In most cases, PAA microcavities with Cv_H_-type cavity layers exhibited superior light confinement properties compared to those with Cv_L_-type cavities. Additionally, shifting the cavity layer from the center toward the edges of the multilayer stack enhanced the intensity of the resonance peak. For PAA microcavities with Cv_H_-type cavity layers, the highest intensity (I_R_ = 53%) and the largest Q-factor (Q = 31) were recorded at λ_C_ of around 5.1 µm. The anodization approach used in this study demonstrates significant potential for designing PAA-based microcavities with high optical performance in the MIR spectral region, especially with further refinement of electrochemical parameters. These findings pave the way for the development of new photonic materials specifically tailored for the MIR spectral range, broadening their applications in various optoelectronic and sensing technologies.

## 1. Introduction

Photonic crystals (PCs) are materials engineered to manipulate the travelling of electromagnetic waves through light-matter interactions [1]. In these structures, the propagation of light is governed by the interference of scattered electromagnetic Bragg waves [2], which can be precisely controlled through the crystal’s structural design. Porous anodic alumina (PAA), produced by anodizing high-purity aluminum foil, has emerged as a promising platform for fabricating one-dimensional photonic crystals (1D PCs) [3,4]. Under carefully optimized electrochemical conditions, the geometry of the pores can be controlled with precision [5,6,7]. By periodically modulating the anodization parameters during pulse anodization, a periodic porous structure is formed, which can be finely tuned to shift photonic stop bands (PSBs) across a wide spectral range [6,7]. PSBs arise from the constructive interference of light reflected at the interfaces between alternating high- and low-porosity (and thus refractive-index) layers. The spectral position and intensity of the PSBs are influenced by factors such as the refractive indices and angles of incidence [8]. Additionally, the bandwidth of reflected light can vary depending on the crystal’s geometry and the refractive index contrast [9].

Various photonic structures based on PAA have been engineered by applying different periodic waveforms of voltage or current density, with variations in shape, amplitude, and anodization mode [10,11,12]. This precise structural modulation significantly enhances PAA’s optical properties [13], making it highly suitable for advanced photonic [3,14] and sensing applications [15,16,17]. Since the optical quality of porous photonic structures strongly depends on variations in morphology, such as layer thickness and porosity, several in situ measuring methods, such as using a laser reflection [18] or photoacoustic techniques [19], have been developed to monitor and adjust processing parameters for improved structural control. Different types of photonic crystals have been fabricated using electrochemical methods, including Fabry–Pérot interferometers [20,21], distributed Bragg reflectors (DBRs) [22,23], gradient-index filters [24,25], and optical microcavities [26,27]. Among these structures, optical microcavities are particularly notable for their ability to trap light within a confined space by inducing electromagnetic wave resonance, allowing light to circulate within the cavity. This confinement enables microcavities to serve as efficient optical amplifiers. One of the key characteristics of optical microcavities is the quality factor (Q-factor), which is a measurement of the cavity’s ability to confine light and can be expressed by the following equation:

(1) $$Q=\frac{\lambda_{c}}{\mathrm{FWHM}}$$

where, λ_c_ refers to the wavelength position of the resonance peak, while FWHM (full width at half maximum) represents the width of the cavity resonance peak at half of its maximum intensity.

Despite ongoing efforts, the development of high-quality PAA microcavities capable of efficiently confining light remains a significant challenge. To date, only a few studies have explored the fabrication of PAA-based optical microcavities with cavity resonance peaks in the visible to near-infrared (VIS-NIR) range [26,27]. These microcavities have been fabricated using various current [26] and voltage [28] profiles, including time-dependent [29], charge-density-dependent [30,31], and optical-path-length-dependent voltage modulation profiles [27,32]. The latter approach achieved the highest reported Q-factor of 269 for the first-order resonance transmission band in the visible spectrum [27]. However, there have been no reports on the fabrication of PAA-based optical microcavities with a cavity resonance peak (λ_C_) in the mid-infrared (MIR) spectral region, even though this range is particularly important for applications such as gas sensing, environmental monitoring, leak detection, and many others [33,34]. The MIR region is highly relevant because many small hazardous gases and molecules, such as CO, CO_2_, NH_4_, and CH_4_, exhibit distinctive absorption fingerprints in this spectral range [35,36]. Developing PAA-based microcavities using straightforward electrochemical processes, combined with their tunable optical properties, could provide a highly efficient and economical solution to advance sensor technologies for detecting these gases.

In this study, we present the first successful fabrication of mid-infrared (MIR) optical microcavities through high-temperature pulse anodization (25 °C) of aluminum in a 0.3 M oxalic acid electrolyte. To position photonic stop bands (PSBs) within the MIR spectrum, a trapezoidal voltage waveform was used to modulate the porosity of alternating high- and low-refractive-index layers in distributed Bragg reflector (DBR) structures. The trapezoidal voltage waveform controls pore formation during anodization by alternating high and low voltages, creating segments of high and low porosity. By shifting the cavity layer from the center toward the edges of the DBR stacks, a spectral shift of the resonant cavity peaks within the 4.5–5.2 μm range was observed. Cavity layers were produced using both low-voltage (Cv_L_) and high-voltage (Cv_H_) pulses, with microcavities featuring Cv_H_-type cavity layers demonstrating superior optical performance. Although the Q-factor values in this initial attempt were lower than those typically observed for PAA-based microcavities in the visible range, our findings clearly indicate that further refinement of the refractive index distribution on both sides of the cavity layers can significantly enhance the optical quality of PAA microcavities in the MIR region. Therefore, these results offer a promising pathway for developing new photonic materials for MIR applications using a simple and cost-effective electrochemical fabrication method.

## 2. Materials and Methods

High-purity aluminum foil (99.9995% Al, Puratronic, Alfa-Aesar, Haverhill, MA, USA) with the dimensions of 20 × 25 × 0.25 mm was used to prepare the PAA microcavities. Before the anodization process, the annealed aluminum specimens were electropolished. Electropolishing was performed in a mixture of 70% concentrated perchloric acid and ethanol at a ratio of 1:4, at 0 °C, under a constant voltage of 25 V for 2.5 min. After the process, the samples were rinsed in ethanol and in distilled water. Next, the electropolished aluminum was anodized in a two-electrode cell. The exposed area of the aluminum sample to the electrolyte solution was 0.96 cm^2^, and the current density was calculated by dividing the input current by the anodized area. The distance between the electrodes aluminum anode and platinum grid cathode was maintained at approximately 3 cm. During the process, a 0.4 L electrochemical Teflon cell was used with a powerful low-temperature constant bath and vigorous stirring at approximately 330 rpm. A programmable DC power supply (Keithley 2614B SourceMeters, Tektronix, Cleveland, OH, USA) was employed to control the electrochemical parameters, including the applied voltage and charge density. The first stage of anodization was carried out under constant voltage of 40 V at 5 °C for 20 h in 0.3 M oxalic acid. The resulting oxide layer was then chemically removed by immersing the samples for 3 h in a mixture of 6 wt% phosphoric acid and 1.8 wt% chromic acid at 65 °C.

In the second stage, pulse anodization was performed in 0.3 M oxalic acid at 25 °C using a trapezoidal pulse profile under charge density-controlled voltage mode. In this mode, the charge density passed during the high (U_H_) and low (U_L_) voltage pulses (C_H_ and C_L_, respectively) remained constant, while the pulse duration varied. For each process, the charge density (the area under the current density curve) was calculated by the program. The U_H_/U_L_ pulse was terminated once the specified target charge density was reached: C_H_ = 2000 mC/cm^2^ for the high-voltage pulse and C_L_ = 2000 mC/cm^2^ for the low-voltage pulse. The resonant cavity layer was created by doubling the charge density for either the U_H_ or U_L_ pulse: Cv_H_ = 2 × C_H_ for the high-voltage cavity layer and Cv_L_ = 2 × C_L_ for the low-voltage cavity layer. Each pulse sequence consisted of four main steps: (1) gradual increase of the voltage from 20 V to 50 V at a ramp rate of 6 V/s, (2) holding the voltage at 50 V until the target charge density (C_H_ or 2 × C_H_ for Cv_H_-type cavity layer) was reached, (3) slow reduction of the voltage from 50 V to 20 V at a rate of 0.234 V/s, (4) continuing anodization at 20 V until the required charge density (C_L_ or 2 × C_L_ for Cv_L_-type cavity layer) was reached. The samples were produced in the configuration “*x* DBR I − Cv_H_/Cv_L_ − *y* DBR II” where *x* and *y* represent the number of cycles applied before and after the Cv_H_/CvL pulses, respectively (with *x* and *y* varying from 14 to 26 cycles, and *x* + *y* = 40 cycles).

The PAA-based photonic structures were studied by a field-emission scanning electron microscope FE-SEM (AMETEK, Inc., Mahwah, NJ, USA). The full thickness of each PAA microcavity was measured using the secondary electron (SE) mode.

The transmission spectra were measured using the Fourier transform infrared spectrometer Alpha II from Bruker Corp., Billerica, MA, USA. Each sample was measured in three spots through a 2 mm diameter diaphragm. The spectra (16 scans per spectrum) were recorded in the range 1.66–25 μm with spectral resolution of 2 cm^−1^. The Gaussian curve fitting for the cavity resonance peaks was performed using Origin software (OriginLab software (OriginLab 2022, OriginLab Corp., Northampton, MA, USA).

## 3. Results and Discussion

**Figure 1:**
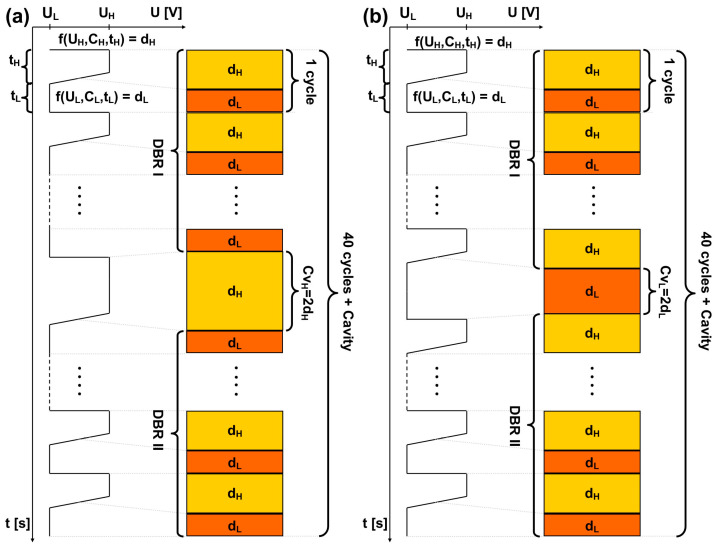
Graphical representations of pulse anodization used to fabricate PAA microcavities, with cavity layers formed under high voltage anodization pulse (Cv_H_) (**a**) and low voltage anodization pulse (Cv_L_) (**b**). U_H_ and U_L_ represent the high and low voltage values, t_H_ and t_L_ denote the duration of the U_H_ and U_L_ pulses respectively, d_H_ and d_L_ correspond to the segment thickness resulting from the anodization under U_H_ and U_L_ pulses, respectively, and C_H_ and C_L_ indicate the charge density passed under the respective U_H_ and U_L_ pulses. The cavity layer is sandwiched between two DBRs, with DBR I being fabricated during the initial stage of the process, before application of the Cv_L_/Cv_H_ pulse, and DBR II formed subsequent to the Cv_L_/Cv_H_ pulse.

To fabricate mid-infrared (MIR) microcavities, we have used a periodic trapezoidal pulse anodizing voltage waveform. Figure 1a,b illustrate the fabrication processes of porous anodic alumina (PAA) microcavities with cavity layers formed under high- (Cv_H_) (a) and low-voltage anodization pulses (Cv_L_) (b). The charge density passing under each successive U_H_ and U_L_ pulse was carefully controlled to allow precise monitoring of the d_H_ and d_L_ layer thickness, respectively. PAA microcavities are designed by placing the Cv_H_ or Cv_L_—type cavity layer (a defective layer) between the top and bottom Distributed Bragg Reflectors (DBR I and DBR II, respectively, as shown in Figure 1). Due to prolonged exposure to the acidic electrolyte, the layers formed during the initial anodization cycles (those constituting DBR I) become more porous than those produced in the later cycles (those forming DBR II). Consequently, even when the number of layers in DBR I is the same as in DBR II, the refractive index distribution, and thus the optical path length, becomes asymmetric on both sides of the cavity. This asymmetry significantly affects the phase of light traveling through the photonic crystal, as well as the bandwidth of the resonance peak resulting from light confinement between the two DBRs. To mitigate the effects of this asymmetry, the number of layers on both sides of the cavity was varied. In other words, the Cv_H_/Cv_L_ cavity layer was positioned between two DBRs with unequal numbers of layers, while keeping the total number of double layers constant at 40.

**Figure 2:**
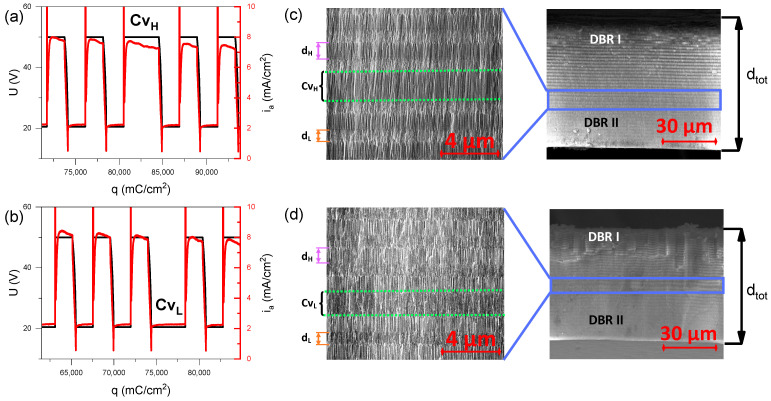
Correlation between anodizing profiles and the morphology of the PAA optical microcavity: Potential pulse sequence U(V), along with the corresponding current density (i_a_) curves as a function of charge density (q) for the Cv_H_ (**a**) and Cv_L_ (**b**) pulses; SEM cross-sectional images of selected PAA microcavities with Cv_H_-type cavity layer (sample 26 DBR I − Cv_H_ − 14 DBR II) (**c**) and Cv_L_-type cavity layer (sample 24 DBR I − Cv_L_ − 16 DBR II) (**d**).

In Figure 2a,b, the U(V) pulse sequence near the Cv_H_ and Cv_L_ pulses is presented, along with the corresponding current density (i_a_) as a function of charge density (q). The modulation of the anodizing voltage results in the formation of a double-layer structure composed of low (d_L_) and high (d_H_) porosity segments, leading to periodic modulation of the refractive index across the PAA. The Cv_H_ and Cv_L_ pulses introduce a defective layer within this periodic structure. Due to the twofold increase in charge density passed during the U_H_ and U_L_ pulses, the Cv_H_-type cavity layer (Figure 2c) is approximately twice as thick as the d_H_ layer, while the Cv_L_-type cavity layer (Figure 2d) is roughly twice as thick as the d_L_ layer. It can be noticed that despite the C_H_ being equal to C_L_, the thickness of the high porosity layer (d_H_~1090 nm) is greater than that of the low porosity layer (d_L_~688 nm). This discrepancy is due to the different efficiencies of oxide growth under high and low anodization voltages, which have been discussed in more detail elsewhere [31,37]. The total thickness (d_tot_) of PAA microcavities is approximately 70 µm. Upon the voltage decrease, small pore branching can be observed, while during the voltage increase, some pores tend to merge due to the increase in interpore distance [38,39]. Various configurations of PAA microcavities were fabricated and studied, which can be categorized into four groups:

I. *x* DBR I − Cv_H_ − *y* DBR II configuration, with x < y
II. *x* DBR I − Cv_L_ − *y* DBR II configuration, with x < y
III. *x* DBR I − Cv_H_ − *y* DBR II configuration, with x > y
IV. *x* DBR I − Cv_L_ − *y* DBR II configuration, with x > y

In all four cases, x + y = 40. The U_H_/U_L_ pulse used to form the Cv_H_/CvL cavity layer was shifted every two cycles earlier (groups 1 and 2) or every two cycles later (groups 3 and 4) with respect to the center of the “20 DBR I − Cv_H_/Cv_L_ − 20 DBR II” pulse sequence.

**Figure 3:**
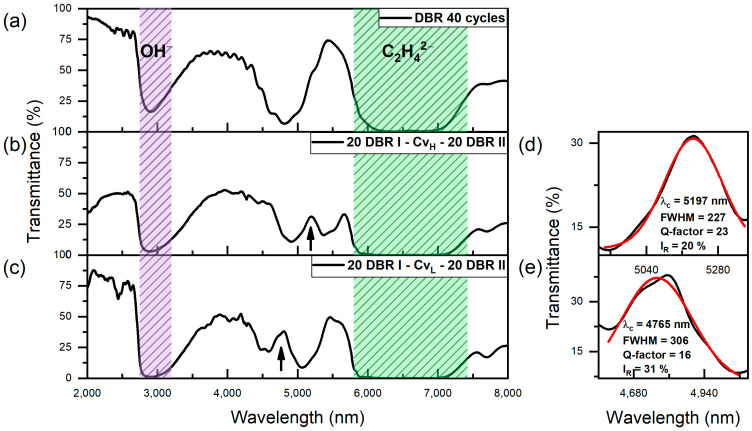
Transmittance spectra of PAA DBR without a defective layer (**a**), PAA microvities with configuration 20 DBR I − Cv_H_ − 20 DBR II (**b**) and 20 DBR I − Cv_L_ − 20 DBR II (**c**). Black arrows indicate the positions of the cavity resonance peaks (λ_c_); Resonanse peak (black line) analysis using a Gaussian function (red line) (**d**,**e**).

Prior to recording the transmittance spectra of the prepared PAA films, the residual Al substrate was selectively dissolved in a saturated aqueous solution of CuCl_2_. Figure 3a shows the transmittance spectrum of the pure PAA-based DBR prepared using a 40-cycle pulse sequence without the C_vH_ or C_vL_ voltage pulses. The photonic structure without a defective layer exhibits a broad first-order photonic stopband (PSB) centered around 4900 nm. In Figure 3b,c, the transmission spectra of samples with configuration of 20 DBR I − Cv_H_ − 20 DBR II and 20 DBR I − Cv_L_ − 20 DBR II (for x = y), respectively, are demonstrated. As can be seen, the insertion of the cavity pulses in the middle of the 40-cycle sequence results in porous structures that possess properties of optical microcavities. The distinct peaks, indicated by black arrows, correspond to the cavity resonance inside the broad first photonic stopbands (PSBs). This demonstrates an actual confinement of mid-infrared light between the two DBR structures. Higher order photonic band gaps are not visible in the transmission spectra. In addition to the PSBs, however, two absorption bands are observed in the transmittance spectra. The band centered around 2770 nm, highlighted in purple, corresponds to the symmetric stretching vibrations of OH− groups coming from adsorbed water molecules in the PAA structure [40]. The second, broader band (5800–7500 nm), marked in green, is attributed to antisymmetric O–C–O bond vibrations, C–C stretching, and O–H deformation vibrations from oxalate impurities derived from oxalic acid [41].

**Figure 4:**
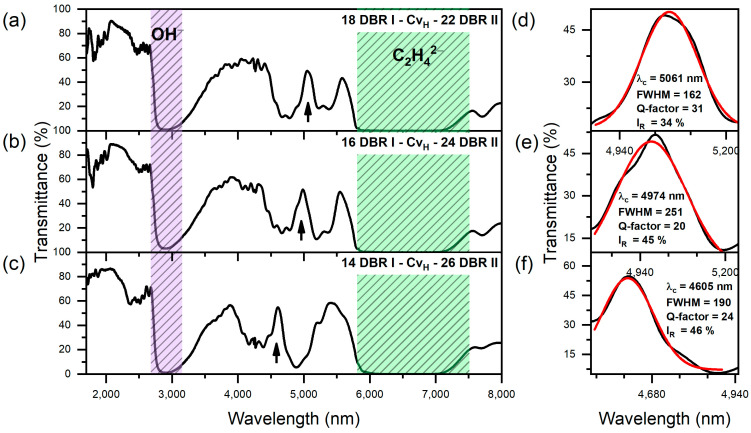
Transmittance spectra of group I PAA microcavities. Black arrows indicate the positions of the cavity resonance peaks (λ_c_) (**a**–**c**); Resonanse peak (black line) analysis using a Gaussian function (red line) (**d**–**f**).

Figure 4a–c show the transmittance spectra of the group I PAA-based microcavities. In Figure 3d,e and Figure 4d–f, the analysis of the cavity resonance peak (λ_c_) is demonstrated using Gaussian fitting. Table 1 presents the data extracted from this fitting, including the spectral position of λ_c_, the full width at half maximum (FWHM) of λ_c_, the resonance peak intensity (I_R_), and the calculated Q-factors. It can be observed that modifying the number of DBR layers on either side of the Cv_H_ cavity significantly affects the resonance peak position, with an increased number of layers in the bottom DBR (DBR II) causing a blue shift in the central wavelength (λ_c_). Specifically, λ_c_ shifts from 5197 nm in the “20 DBR I − Cv_H_ − 20 DBR II” sample (Figure 3b,d) to 4605 nm in the “14 DBR I − Cv_H_ − 26 DBR II” sample (Figure 4c,f). In addition to this wavelength shift, the resonance intensity (I_R_) varies with the asymmetry in DBR configurations. The I_R_ values increase as the number of DBR II layers increases at the expense of the layers in DBR I. The “20 DBR I − Cv_H_ − 20 DBR II” sample exhibits the lowest intensity at 20%, whereas the “14 DBR I − Cv_H_ − 26 DBR II” sample reaches the highest resonance intensity at 46%. Similarly, a clear decrease in FWHM of the λ_C_ band is observed as “x” increases while “y” decreases simultaneously (Table 1). This trend indicates that asymmetric PAA microcavity configurations, where one side DBR has more layers than the other, help to balance the optical characteristics of the DBR I and DBR II, enhance optical confinement, and improve the efficiency of light coupling into the cavity. For the group I PAA-based microcavities, the highest Q-factor of 31 at λ_C_ = 5061 nm was achieved in the “18 DBR I − Cv_H_ − 22 DBR II” configuration. It is worth mentioning that this Q-factor is higher than the value of 24 obtained in PAA microcavities fabricated using a similar pulse sequence in a 0.3 M oxalic acid electrolyte, where λ_C_ was located at 953 nm [28]. For the other structures, Q-factors ranged between 20 and 24. This variability can likely be attributed to differences in the structural quality of the porous layers, particularly those most exposed to the acidic electrolyte, i.e., those included in DBR I.

**Figure 5:**
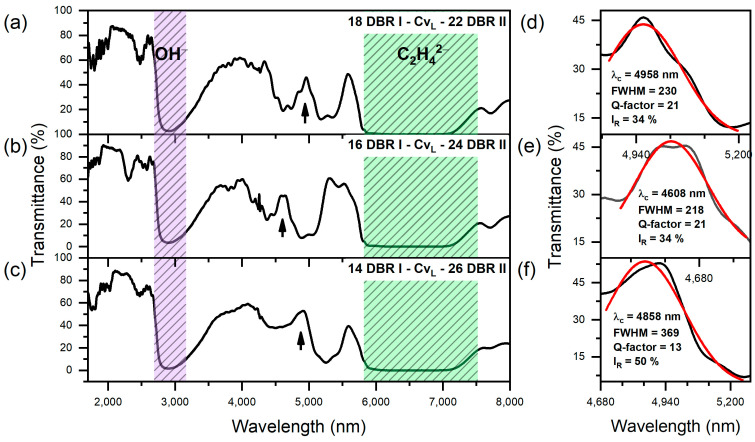
Transmittance spectra of group II PAA microcavities. Black arrows indicate the positions of the cavity resonance peaks (λ_c_) (**a**–**c**); Resonanse peak (black line) analysis using a Gaussian function (red line) (d–**f**).

The transmittance spectra of group II PAA microcavities are shown in Figure 5, along with the Gaussian analysis of the resonance peak (λ_C_). In Table 1, the spectral position, full width at half maximum (FWHM), resonance intensity (I_R_), and Q-factor of the resonance peak (λ_C_) for these samples are listed. First, it can be noticed that the shape of the λ_C_ bands is more irregular compared to those recorded in group I PAA microcavities. The central position of λ_C_ fluctuates across the samples, ranging from 4608 nm in the “16 DBR I − Cv_L_ − 24 DBR II” sample to 4958 nm in the “18 DBR I − Cv_L_ − 22 DBR II” sample. Additionally, there is a noticeable increase in resonance intensity as the number of layers in DBR II increases, indicating improved optical performance with a greater number of DBR II layers. The highest resonance intensity, 50%, is observed in the “14 DBR I − Cv_L_ − 26 DBR II” sample (Figure 5c,f), while the “20 DBR I − Cv_L_ − 20 DBR II” sample (Figure 3c,e) shows the lowest resonance intensity (I_R_) at 31%. As in group I PAA microcavities, the FWHM tends to decrease with the reduction of x, except for the sample with the lowest x (“14 DBR I − Cv_L_ − 26 DBR II”), where the FWHM reaches its highest value. The Q-factor exhibits moderate variation across the samples, with the highest values of 21 recorded for the “18 DBR I − Cv_L_ − 22 DBR II” and “16 DBR I − Cv_L_ − 24 DBR II” (Table 1) samples. The lowest Q-factor of 13, along with the largest FWHM value of 369, observed in the “14 DBR I − Cv_L_ − 26 DBR II” sample (Figure 5c,f), may be attributed to the severe damage of the porous segments and the loss of structural integrity in DBR I. This photonic structure was designed to consist of only 14 double layers, many of which were likely dissolved during the synthesis process due to prolonged interaction with the oxalic acid solution. As a result, the reduced number of layers in DBR I likely weakened the optical confinement, resulting in the weakest signal from the cavity and consequently the lowest Q-factor. This loss of optical integrity suggests that maintaining a sufficient number of DBR I layers is crucial for enhancing the performance of the microcavity. Moreover, it can be observed that the optical quality of PAA microcavities with the Cv_L_ layer is lower than that of those with the Cv_H_ layer. This indicates that light is better confined when the cavity layer, characterized by lower porosity and a higher refractive index (Cv_H_ = 2 × d_H_ layer), is positioned directly adjacent to layers with higher porosity and, consequently, lower refractive index (d_L_ layers). This configuration enhances optical confinement within the cavity, contributing to better optical performance.

**Figure 6:**
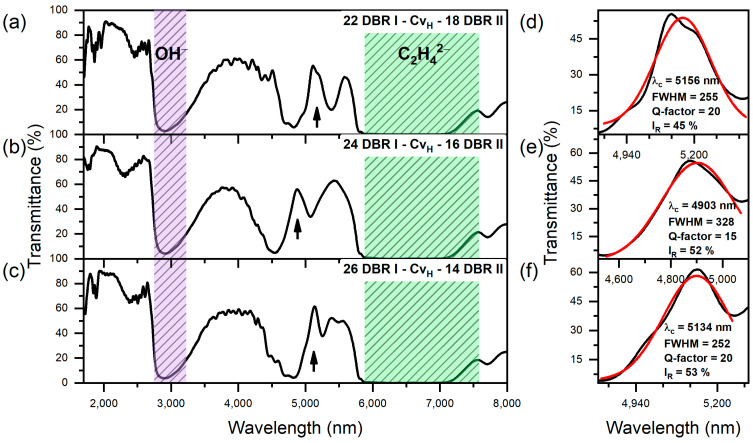
Transmittance spectra of group III PAA microcavities. Black arrows indicate the positions of the cavity resonance peaks (λ_c_) (**a**–**c**); Resonanse peak (black line) analysis using a Gaussian function (red line) (**d**–**f**).

To maintain a sufficient number of layers in DBR I, the group III and IV PAA microcavity with the with x > y were prepared. In Figure 6, transmittance spectra of PAA microcavities from the group III are shown, along with the Gaussian fitting of the cavity resonance peak λ_c_. Table 1 presents the spectral position, FWHM, I_R_, and Q-factor of the λ_c_ for each sample, as extracted from the Gaussian fitting (Figure 6d–f). Unlike the PAA microcavities where DBR I contains fewer double layers than DBR II, the spectral position of the λ_c_ peak in these samples does not exhibit a significant shift compared to the “20 DBR I − Cv_H_ − 20 DBR II” sample. However, the resonance intensity (I_R_) increases substantially, from 20% in the “20 DBR I − Cv_H_ − 20 DBR II” (Figure 3d) to 53% in the “26 DBR I − Cv_H_ − 14 DBR II” microcavity (Figure 6f). Despite this increase in I_R_, the Q-factor does not exceed 20 in these photonic configurations, indicating that increasing the number of DBR I layers did not enhance the optical quality of the PAA microcavities in the MIR spectral region. This suggests that the additional DBR I layers primarily improve the resonance intensity rather than the overall optical confinement or sharpness of the resonance peak.

**Figure 7:**
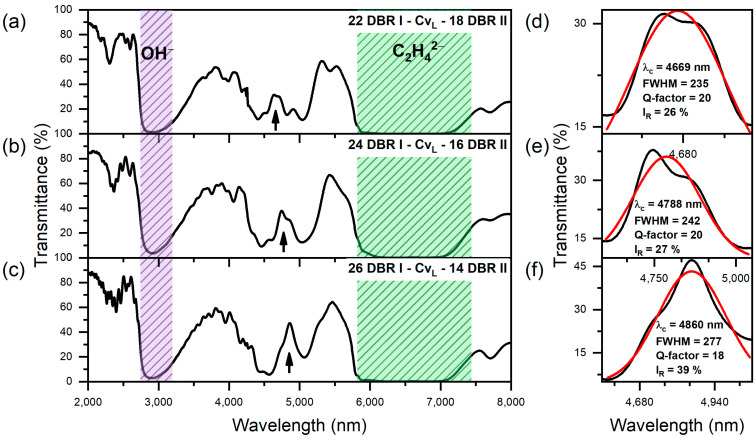
Transmittance spectra of group IV PAA microcavities. Black arrows indicate the positions of resonance peaks (λ_c_) (**a**–**c**); Resonanse peak (black line) analysis using a Gaussian function (red line) (**d**–**f**).

In Figure 7, the transmittance spectra of the group IV PAA microcavities, along with the Gaussian analysis of the λ_C_ band, are presented. The optical parameters derived from the Gaussian fit to the cavity resonance peak (λ_C_) are gathered in Table 1. The data demonstrate a modest improvement in the Q-factor values for the “22 DBR I − Cv_L_ − 18 DBR II” (Q = 20), “24 DBR I − Cv_L_ − 16 DBR II” (Q = 20), and “26 DBR I − Cv_L_ − 14 DBR I” (Q = 22) samples, compared to the “20 DBR I − Cv_L_ − 20 DBR II” sample, which had a Q-factor of 16. The spectral position λ_C_ also exhibits a slight red shift, moving from 4765 nm in the “20 DBR I − Cv_L_ − 20 DBR II” microcavity to 4860 nm in the “26 DBR I − Cv_L_ − 14 DBR II” microcavity. However, similar to the microcavities with the Cv_H_-type cavity layer, increasing the number of double layers in DBR I at the expense of those in DBR II does not significantly enhance the overall optical performance of PAA-based microcavities in the MIR region. When comparing optical parameters, such as FWHM and Q-factors, between groups III and IV (Table 1), no definitive conclusion can be drawn regarding which group demonstrates a better tendency to confine mid-infrared (MIR) light. Yet, it is important to remember that these parameters are affected by the Gaussian fitting process itself, which is not always optimal, particularly in cases of very irregular λ_C_ peak shapes. However, the transmission spectra from group III (Figure 6) and group IV (Figure 7) suggest that PAA microcavities with the Cv_H_-type cavity layer exhibit superior MIR light confinement properties. These are characterized by enhanced intensity and more regular resonance peaks compared to those recorded for the PAA microcavities with a Cv_L_-type cavity layer, regardless of its position within the multilayer stack.

Summarizing the results, the optical quality of PAA-based microcavities is strongly influenced by the effective refractive index (*n_eff_*) of the distributed Bragg reflectors (DBRs) on both sides of the cavity layer. Symmetry in *n_eff_* can be further enhanced by carefully designing the pulse sequence to achieve a uniform distribution of optical thickness (d_opt_ = d_i_ · n_i_, where *i* is the number of a given layer) for the alternating low (d_H_) and high (d_L_) porosity layers in both DBR I and DBR II. While the charge density-controlled voltage mode allows precise control over the physical thickness (d_i_) of alternating segments, special attention must be given to adjusting this thickness to account for variations in porosity across the PAA structure, ensuring a constant d_opt_ value. Introducing variability in the thickness of the low and high porosity layers can significantly improve the Q-factors in MIR PAA microcavities, which is crucial for specific mid-infrared applications. This way, it will be possible to obtain microcavities with better optical quality and customized resonance properties to meet specific technological needs. Further work is ongoing to optimize this process.

## 4. Conclusions

In this work, we present a first report on the production of mid-infrared (MIR) porous anodic alumina (PAA)-based microcavities with tunable optical quality. The spectral position of the cavity resonance peak, along with its intensity and Q-factor, is shown to vary depending on the geometric position of the cavity layer within the multilayer stack as well as the type of cavity produced—either by high voltage (Cv_H_-type cavity) or low voltage (Cv_L_-type cavity) pulses. In most of the cases studied, PAA microcavities with Cv_H_-type cavity layers demonstrated superior light confinement properties compared to those with Cv_L_-type cavity layers. Additionally, shifting the cavity layer from the center towards the edges of the multilayer stack was found to enhance the intensity of the resonance wavelength (λ_C_). The highest intensity (I_R_) of approximately 53% was achieved in the 26-DBR I − Cv_H_ − 14-DBR II microcavity configuration. The optimal microcavity design (18-DBR I − Cv_H_ − 22-DBR II) exhibited a Q-factor of 31. While these Q-factor values are lower than those typically obtained in PAA-based microcavities with λ_C_ in the visible spectral range, our study demonstrates the feasibility of designing PAA-based microcavities with good optical performance in the MIR spectral region. Additionally, by leveraging the tunable light-confining properties of PAA microcavities, along with their chemical and mechanical robustness, PAA-based photonic structures could broaden their applicability in fields that require high optical sensitivity in the mid-infrared range, low-cost production, and environmental durability.

## Figures and Tables

**Table 1:** Spectral position, full width at half maximum (FWHM), resonance intensity (I_R_) and Q-factor of the cavity resonance peak (λ_c_).

	Sample	λ_C_ (nm)	FWHM	Resonance Intensity IR (%)	Q-Factor
1.	20 DBR I − CvH − 20 DBR II	5197	227	20	23
2.	18 DBR I − CvH − 22 DBR II	5061	162	34	31
3.	16 DBR I − CvH − 24 DBR II	4974	251	45	20
4.	14 DBR I − CvH − 26 DBR II	4605	190	46	24
5.	20 DBR I − CvL − 20 DBR II	4765	306	31	16
6.	18 DBR I − CvL − 22 DBR II	4958	230	34	21
7.	16 DBR I − CvL − 24 DBR II	4608	218	34	21
8.	14 DBR I − CvL − 26 DBR II	4858	369	50	13
9.	22 DBR I − CvH − 18 DBR II	5156	255	45	20
10.	24 DBR I − CvH − 16 DBR II	4903	328	52	15
11.	26 DBR I − CvH − 14 DBR II	5134	252	53	20
12.	22 DBR I − CvL − 18 DBR II	4669	235	26	20
13.	24 DBR I − CvL − 16 DBR II	4788	242	27	20
14.	26 DBR I − CvL − 14 DBR II	4860	277	39	18

## Data Availability

The original contributions presented in this study are included in the article. Further inquiries can be directed to the corresponding author.
